# Investigation of the Interaction between Cdc42 and Its Effector TOCA1: HANDOVER OF Cdc42 TO THE ACTIN REGULATOR N-WASP IS FACILITATED BY DIFFERENTIAL BINDING AFFINITIES*

**DOI:** 10.1074/jbc.M116.724294

**Published:** 2016-04-22

**Authors:** Joanna R. Watson, Helen M. Fox, Daniel Nietlispach, Jennifer L. Gallop, Darerca Owen, Helen R. Mott

**Affiliations:** From the ‡ Department of Biochemistry, 80 Tennis Court Road, University of Cambridge, Cambridge CB2 1GA and; the § Wellcome Trust/Cancer Research UK Gurdon Institute, University of Cambridge, Cambridge CB2 1QN, United Kingdom

**Keywords:** actin, CDC42, endocytosis, nuclear magnetic resonance (NMR), protein-protein interaction, BAR domain, CIP4, FBP17, TOCA1, WASP

## Abstract

Transducer of Cdc42-dependent actin assembly protein 1 (TOCA1) is an effector of the Rho family small G protein Cdc42. It contains a membrane-deforming F-BAR domain as well as a Src homology 3 (SH3) domain and a G protein-binding homology region 1 (HR1) domain. TOCA1 binding to Cdc42 leads to actin rearrangements, which are thought to be involved in processes such as endocytosis, filopodia formation, and cell migration. We have solved the structure of the HR1 domain of TOCA1, providing the first structural data for this protein. We have found that the TOCA1 HR1, like the closely related CIP4 HR1, has interesting structural features that are not observed in other HR1 domains. We have also investigated the binding of the TOCA HR1 domain to Cdc42 and the potential ternary complex between Cdc42 and the G protein-binding regions of TOCA1 and a member of the Wiskott-Aldrich syndrome protein family, N-WASP. TOCA1 binds Cdc42 with micromolar affinity, in contrast to the nanomolar affinity of the N-WASP G protein-binding region for Cdc42. NMR experiments show that the Cdc42-binding domain from N-WASP is able to displace TOCA1 HR1 from Cdc42, whereas the N-WASP domain but not the TOCA1 HR1 domain inhibits actin polymerization. This suggests that TOCA1 binding to Cdc42 is an early step in the Cdc42-dependent pathways that govern actin dynamics, and the differential binding affinities of the effectors facilitate a handover from TOCA1 to N-WASP, which can then drive recruitment of the actin-modifying machinery.

## Introduction

The Ras superfamily of small GTPases comprises over 150 members that regulate a multitude of cellular processes in eukaryotes. The superfamily can be divided into five families based on structural and functional similarities: Ras, Rho, Rab, Arf, and Ran. All members share a well defined core structure of ∼20 kDa known as the G domain, which is responsible for guanine nucleotide binding (1). It is this guanine nucleotide binding that underlies their function as molecular switches, controlling a vast array of signaling pathways. These molecular switches cycle between active, GTP-bound, and inactive, GDP-bound, states with the help of auxiliary proteins. The guanine nucleotide exchange factors mediate formation of the active state by promoting the dissociation of GDP, allowing GTP to bind. The GTPase-activating proteins stimulate the rate of intrinsic GTP hydrolysis, mediating the return to the inactive state (reviewed in Ref. 2).

The overall conformation of small G proteins in the active and inactive states is similar, but they differ significantly in two main regions known as switch I and switch II. These regions are responsible for “sensing” the nucleotide state, with the GTP-bound state showing greater rigidity and the GDP-bound state adopting a more relaxed conformation (reviewed in Ref. 3). In the active state, G proteins bind to an array of downstream effectors, through which they exert their extensive roles within the cell. The structures of more than 60 small G protein·effector complexes have been solved, and, not surprisingly, the switch regions have been implicated in a large proportion of the G protein-effector interactions (reviewed in Ref. 4). However, because each of the 150 members of the superfamily interacts with multiple effectors, there are still a huge number of known G protein-effector interactions that have not yet been studied structurally.

The Rho family comprises 20 members, of which three, RhoA, Rac1, and Cdc42, have been relatively well studied. The role of these three proteins in the coordination of the actin cytoskeleton has been examined extensively (5–10). RhoA acts to rearrange existing actin structures to form stress fibers, whereas Rac1 and Cdc42 promote *de novo* actin polymerization to form lamellipodia and filopodia, respectively (9–12). A number of RhoA and Rac1 effector proteins, including the formins (13) and members of the protein kinase C-related kinase (PRK)^6^ family (14), along with Cdc42 effectors, including the Wiskott-Aldrich syndrome (WASP) family (15) and the transducer of Cdc42-dependent actin assembly (TOCA) family (16–18), have also been linked to the pathways that govern cytoskeletal dynamics.

Cdc42 effectors, TOCA1 and the ubiquitously expressed member of the WASP family, N-WASP, have been implicated in the regulation of actin polymerization downstream of Cdc42 and phosphatidylinositol 4,5-bisphosphate (PI(4,5)P_2_) (9, 16, 19–22). N-WASP exists in an autoinhibited conformation, which is released upon PI(4,5)P_2_ and Cdc42 binding (21, 23) or by other factors, such as phosphorylation (24). Following their release, the C-terminal regions of N-WASP are free to interact with G-actin and a known nucleator of actin assembly, the Arp2/3 complex (25). The importance of TOCA1 in actin polymerization has been demonstrated in a range of *in vitro* and *in vivo* studies (16, 26–32), but the exact role of TOCA1 in the many pathways involving actin assembly remains unclear. The most widely studied role of TOCA1 is in membrane invagination and endocytosis (28–30, 33, 34), although it has also been implicated in filopodia formation (27), neurite elongation (35), transcriptional reprogramming via nuclear actin (36), and interaction with ZO-1 at tight junctions (37). A role in cell motility and invasion has also been established (38, 39).

TOCA1 comprises an N-terminal F-BAR domain, a central homology region 1 (HR1) domain, and a C-terminal SH3 domain. The F-BAR domain is a known dimerization, membrane-binding, and membrane-deforming module (33, 40, 41) found in a number of cell signaling proteins. The TOCA1 SH3 domain has many known binding partners, including N-WASP (16) and dynamin (40). The HR1 domain has been directly implicated in the interaction between TOCA1 and Cdc42 (16), representing the first Cdc42-HR1 domain interaction to be identified.

Other HR1 domains studied so far, including those from the PRK family, have been found to bind their cognate Rho family G protein-binding partner with high specificity and affinities in the nanomolar range (42–45). The structures of the PRK1 HR1a domain in complex with RhoA (42) and the HR1b domain in complex with Rac1 (46) show that the HR1 domain comprises an anti-parallel coiled-coil that interacts with its G protein binding partner via both helices. Both of the G protein switch regions are involved in the interaction. The coiled-coil fold is shared by the HR1 domain of the TOCA family protein, CIP4 (47), and, based on sequence homology, by TOCA1 itself. These HR1 domains, however, show specificity for Cdc42, rather than RhoA or Rac1 (16, 17). How different HR1 domain proteins distinguish their specific G protein partners remains only partially understood, and structural characterization of a novel G protein-HR1 domain interaction would add to the growing body of information pertaining to these protein complexes. Furthermore, the biological function of the interaction between TOCA1 and Cdc42 remains poorly understood, and so far there has been no biophysical or structural insight.

The interactions of TOCA1 and N-WASP with Cdc42 as well as with each other have raised questions as to whether the two Cdc42 effectors can interact with a single molecule of Cdc42 simultaneously. There is some evidence for a ternary complex between Cdc42, N-WASP, and TOCA1 (30), but there was no direct demonstration of simultaneous contacts between the two effectors and a single molecule of Cdc42. Nonetheless, the substantial difference between the structures of the G protein-binding regions of the two effectors is intriguing and implies that they bind to Cdc42 quite differently, providing motivation for investigating the possibility that Cdc42 can bind both effectors concurrently. WASP interacts with Cdc42 via a conserved, unstructured binding motif known as the Cdc42- and Rac-interactive binding region (CRIB) (48), which forms an intermolecular β-sheet, expanding the anti-parallel β2 and β3 strands of Cdc42 (49). In contrast, the TOCA family proteins are thought to interact via the HR1 domain, which may form a triple coiled-coil with switch II of Rac1, like the HR1b domain of PRK1 (46).

Here, we present the solution NMR structure of the HR1 domain of TOCA1, providing the first structural data for this protein. We also present data pertaining to binding of the TOCA HR1 domain to Cdc42, which is the first biophysical description of an HR1 domain binding this particular Rho family small G protein. Finally, we investigate the potential ternary complex between Cdc42 and the G protein-binding regions of TOCA1 and N-WASP, contributing to our understanding of G protein-effector interactions as well as the roles of Cdc42, N-WASP, and TOCA1 in the pathways that govern actin dynamics.

## Experimental Procedures

### 

#### 

##### Expression Constructs

The *Xenopus tropicalis* TOCA1 HR1 domain (residues 330–426 and N-terminally extended constructs as indicated) were amplified from cDNA (TOCA1 accession number NM_001005148) and cloned into pGEX-6P-1 (GE Healthcare) or pGEX-HisP (44). The HR1 domain of human CIP4 (residues 388–481) was amplified from IMAGE clone 3532036, the *Xenopus laevis* FBP17 HR1 domain (residues 385–486) from IMAGE clone 5514481, and the *X. tropicalis* N-WASP G protein-binding domain (GBD) (residues 197–255) from IMAGE clone 5379332, and all were cloned into pGEX-6P-1. The resulting constructs express the proteins as N-terminal GST fusions with a 3C protease-cleavable tag, with pGEX-HisP expressing an additional C-terminal His_6_ tag. Human Cdc42Δ7Q61L and full-length Cdc42 were cloned into pGEX-2T (GE Healthcare) and pGEX-6P-1, respectively. A C-terminally extended construct of TOCA1 comprising residues 330–545 was cloned into pMAT10-P.^7^ The resulting construct expresses TOCA1 330–545 as an N-terminal His-MBP fusion protein with a 3C protease-cleavable tag. Full-length *X. tropicalis* TOCA1, TOCA1 F-BAR (residues 1–287), and TOCA1 ΔSH3 (residues 1–480) were PCR-amplified from a cDNA clone (IMAGE 5157175) and cloned into pET-His_6_-SNAP using FseI and AscI sites that had been incorporated into the primers to create His-SNAP-TOCA1 proteins.

##### Protein Expression

GST fusion proteins (HR1 domains and Cdc42) were expressed in *E. coli* BL21 cells (Invitrogen). Stationary cultures were diluted 1:10 and grown at 37 °C until an *A*_600_ of ∼0.8 was reached and then induced with 0.1 mm isopropyl-β-d-thiogalactopyranoside for 20 h at 20 °C. The GST-N-WASP GBD construct was expressed in *E. coli* BL21-CodonPlus®-RIL (Agilent Technologies). The proteins were purified using glutathione-agarose beads (Sigma) and eluted from the beads by cleavage of the GST tag with 3C protease (HR1 domains, N-WASP GBD, and full-length Cdc42Q61L) or thrombin (Novagen, Cdc42Δ7Q61L) prior to gel filtration on a 16/60 S75 column (GE Healthcare). His-MBP-HR1-SH3 was purified using nickel-nitrilotriacetic acid-agarose beads (Life Technologies) prior to cleavage with 3C protease and gel filtration. Full-length TOCA1, TOCA1 F-BAR, and TOCA1 ΔSH3 were expressed from pET-His_6_-SNAP in BL21 pLysS, grown at 37 °C until an *A*_600_ of ∼0.6 was reached, and induced with 0.3 mm isopropyl-β-d-thiogalactopyranoside overnight at 19 °C. Proteins were coupled to nickel-nitrilotriacetic acid-agarose (Qiagen), eluted using increasing concentrations of imidazole, and further purified by gel filtration using a 16/60 S200 column (GE Healthcare). All protein concentrations were determined by amino acid analysis (Protein and Nucleic Acid Chemistry Facility, Department of Biochemistry, University of Cambridge).

##### Nucleotide Exchange

For NMR experiments, Cdc42 was nucleotide-exchanged for the non-hydrolyzable GTP analogue GMPPNP (Sigma) as described previously (50). For scintillation proximity assays (SPAs), Cdc42 was loaded with [^3^H]GTP using [8-^3^H]GTP (PerkinElmer Life Sciences), as described previously (51). The protein was confirmed as full-length using mass spectrometry (PNAC facility, Department of Biochemistry, University of Cambridge).

##### SPAs

For direct assays, GST-PAK, GST-ACK, or His-tagged TOCA1 constructs were attached to a fluoromicrosphere via an anti-GST or anti-His antibody in the presence of Cdc42Δ7Q61L·[^3^H]GTP. Binding curves were fitted using a direct binding isotherm to obtain *K_d_* values and their curve-fitting errors for the G protein-effector interactions (52). For competition assays, free ACK GBD, TOCA1 HR1, TOCA1 HR1SH3, or N-WASP GBD was titrated into a mixture of 30 nm Cdc42Δ7Q61L·[^3^H]GTP and 30 nm GST-ACK immobilized on a fluoromicrosphere as above. Data were fitted to competition binding isotherms to obtain *K_d_* values and curve-fitting errors, as described previously (53).

##### NMR Spectroscopy

The NMR experiments and resonance assignments of the HR1 domain are described (54). The NMR experiments were carried out with 0.9 mm ^13^C/^15^N-labeled HR1 domain in 20 mm sodium phosphate, pH 7.5, 150 mm NaCl, 5 mm MgCl_2_, 5 mm DTT, 10% D_2_O. Distance restraints were derived from a ^15^N-separated NOESY (100-ms mixing time) recorded on a Bruker DRX500 and a ^13^C-separated NOESY (100-ms mixing time) recorded on an Avance AV600. NMR data were processed using *AZARA* (W. Boucher, University of Cambridge) and analyzed using *ANALYSIS* (55).

##### Structure Calculation

Structures were calculated iteratively using CNS version 1.0 interfaced to Aria version 2.3.1 (56). The PROSLQ force field was used for non-bonded parameters. Backbone torsion angles were estimated from CA, CO, CB, N, and HA chemical shifts using TALOS-N (57). The “strong” φ and ψ restraints were included with an error of ±2 S.D. values of the averaged TALOS-N predictions. Dihedral angle predictions for residues 323–340 were weak, so no restraints were included for this region.

##### NMR Titrations

All of the ^15^N and ^13^C HSQCs were recorded at 25 °C in 50 mm sodium phosphate, pH 5.5, 25 mm NaCl, 5 mm MgCl_2_, 5 mm DTT, 10% D_2_O on a Bruker DRX500. ^15^N-HR1 HSQC experiments were recorded on 0.2 mm ^15^N-HR1 domain with HR1/Cdc42·GMPPNP ratios of 1:0, 1:0.25, 1:0.5, 1:1, and 1:4. Experiments were recorded on 0.27 mm ^15^N-Cdc42·GMPPNP at Cdc42/HR1 ratios of 1:0, 1:0.25, 1:0.5, and 1:2.2. The ^15^N HSQC titrations with N-WASP were recorded on 0.6 mm ^15^N-HR1 domain or 0.15 mm ^15^N-Cdc42 at the ratios indicated in the figures.

##### Chemical Shift Mapping

The chemical shift changes, δ, were calculated using the equation,

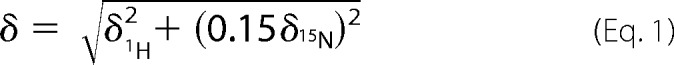
 where δ(^1^H) and δ(^15^N) are the chemical shift changes for the ^1^H and ^15^N dimensions, respectively. Residues that had disappeared were assigned a δ value larger than the maximum calculated δ for the data set, and residues that were too overlapped to be reliably assigned in the complex spectra were assigned δ = 0. The residues that had shifted more than the mean chemical shift change across the spectrum were classed as significant and were filtered for solvent accessibility using *NACCESS* (58). Residues with <50% solvent accessibility were considered to be buried and unavailable for binding.

##### Pyrene Actin Assays

Pyrene actin assays were carried out as described previously (59). *Xenopus* high speed supernatant was used at 5 mg/ml and supplemented with 0.12 mg/ml pyrene actin as described previously (32). TOCA1 HR1 domain or N-WASP CRIB domain was added at the concentrations indicated. Liposomes were made, using methods described previously (60), from 60% phosphatidylcholine, 30% phosphatidylserine, and 10% PI(4,5)P_2_ to 2 mm final lipid concentration. All of the lipids used were natural brain or liver lipids from Avanti Polar Lipids. The assays were initiated by the addition of 5 μl of liposomes per 200 μl of reaction mix.

## Results

### 

#### 

##### Cdc42-TOCA1 Binding

TOCA1 was identified in *Xenopus* extracts as a protein necessary for Cdc42-dependent actin assembly (16) and was shown to bind to Cdc42·GTPγS but not to Cdc42·GDP or to Rac1 and RhoA. Given its homology to other Rho family binding modules, it is likely that the HR1 domain of TOCA1 is sufficient to bind Cdc42. The *C. elegans* TOCA1 orthologues also bind to Cdc42 via their consensus HR1 domain (34). The HR1 domains from the PRK family bind their G protein partners with a high affinity, exhibiting a range of submicromolar dissociation constants (*K_d_*) as low as 26 nm (45). A *K_d_* in the nanomolar range was therefore expected for the interaction of the TOCA1 HR1 domain with Cdc42.

**FIGURE 1.**
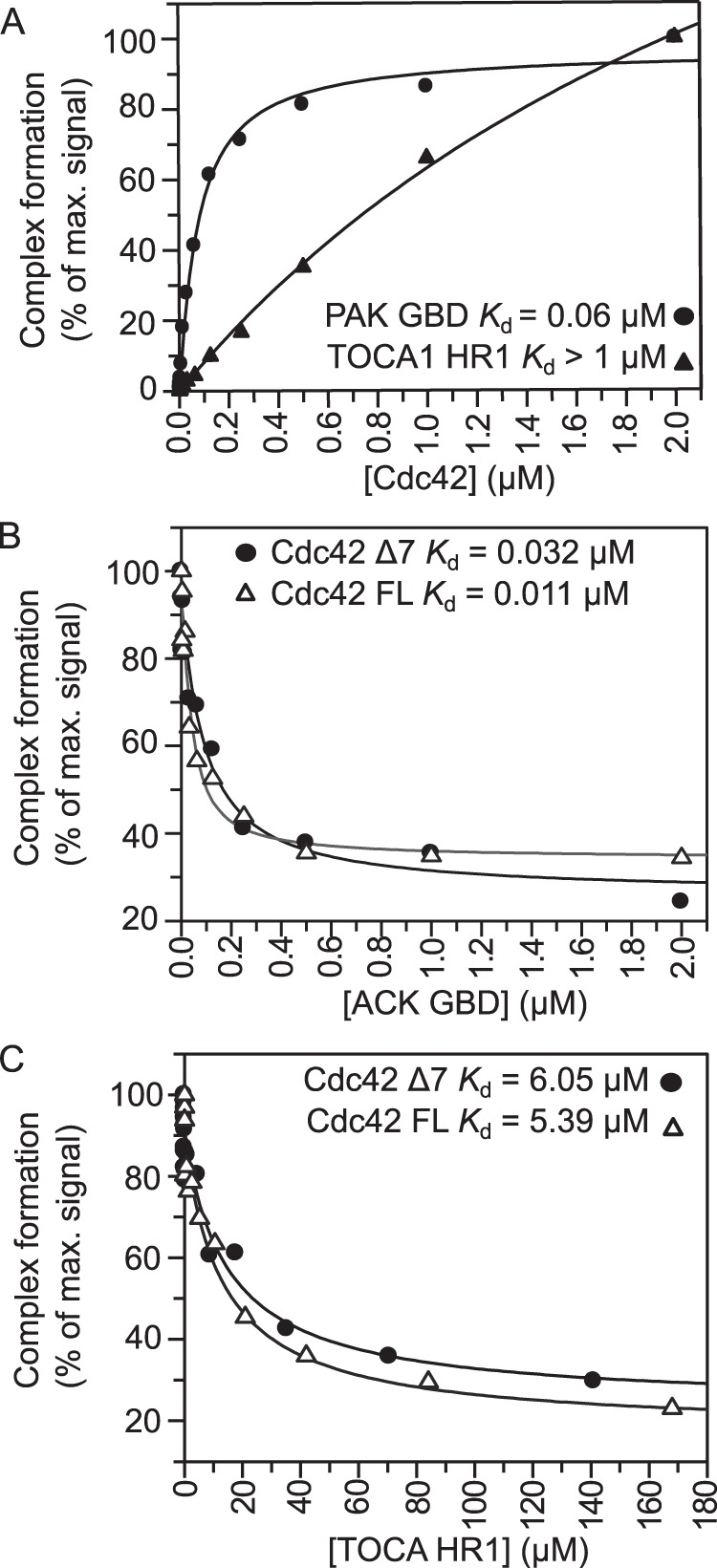
**The TOCA1 HR1-Cdc42 interaction is low affinity.** *A*, curves derived from direct binding assays in which the indicated concentrations of Cdc42Δ7Q61L·[^3^H]GTP were incubated with 30 nm GST-PAK or HR1-His_6_ in SPAs. The SPA signal was corrected by subtraction of control data with no GST-PAK or HR1-His_6_. The data were fitted to a binding isotherm to give an apparent *K_d_* and are expressed as a percentage of the maximum signal; *B* and *C*, competition SPA experiments were carried out with the indicated concentrations of ACK GBD (*B*) or HR1 domain (*C*) titrated into 30 nm GST-ACK and either 30 nm Cdc42Δ7Q61L·[^3^H]GTP or full-length Cdc42Q61L·[^3^H]GTP. The *K_d_* values derived for the ACK GBD with Cdc42Δ7 and full-length Cdc42 were 0.032 ± 0.01 and 0.011 ± 0.01 μm, respectively. The *K_d_* values derived for the TOCA1 HR1 with Cdc42Δ7 and full-length Cdc42 were 6.05 ± 1.96 and 5.39 ± 1.69 μm, respectively.

We generated an *X. tropicalis* TOCA1 HR1 domain construct encompassing residues 330–426. This region comprises the complete HR1 domain based on secondary structure predictions and sequence alignments with another TOCA family member, CIP4, whose structure has been determined (47). The interaction between [^3^H]GTP·Cdc42 and a C-terminally His-tagged TOCA1 HR1 domain construct was investigated using SPA. The binding isotherm for the interaction is shown in Fig. 1*A*, together with the Cdc42-PAK interaction as a positive control (52). The binding of TOCA1 HR1 to Cdc42 was unexpectedly weak, with a *K_d_* of >1 μm. It was not possible to estimate the *K_d_* more accurately using direct SPA experiments, because saturation could not be reached due to nonspecific signal at higher protein concentrations.

It was possible that the low affinity observed was due to negative effects of immobilization of the HR1 domain, so other methods were employed, which utilized untagged proteins. Isothermal titration calorimetry was carried out, but no heat changes were observed at a range of concentrations and temperatures (data not shown), suggesting that the interaction is predominantly entropically driven. Other G protein-HR1 domain interactions have also failed to show heat changes in our hands.^7^ Infrared interferometry with immobilized Cdc42 was also attempted but was unsuccessful for both TOCA1 HR1 and for the positive control, ACK.

The affinity was therefore determined using competition SPAs. A complex of a GST fusion of the GBD of ACK, which binds with a high affinity to Cdc42 (61), with radiolabeled [^3^H]GTP·Cdc42 was preformed, and the effect of increasing concentrations of untagged TOCA1 HR1 domain was examined. Competition of GST-ACK GBD bound to [^3^H]GTP·Cdc42 by free ACK GBD was used as a control and to establish the value of background counts when Cdc42 is fully displaced. The data were fitted to a binding isotherm describing competition (53). Free ACK competed with itself with an affinity of 32 nm, similar to the value obtained by direct binding of 23 nm (61). The TOCA1 HR1 domain also fully competed with the GST-ACK but bound with an affinity of 6 μm (Fig. 1, *B* and *C*), in agreement with the low affinity observed in the direct binding experiments.

The Cdc42 construct used in the binding assays has seven residues deleted from the C terminus to facilitate purification. These residues are not generally required for G protein-effector interactions, including the interaction between RhoA and the PRK1 HR1a domain (53). In contrast, the C terminus of Rac1 contains a polybasic sequence, which is crucial for Rac1 binding to the HR1b domain from PRK1 (43, 46). As the observed affinity between TOCA1 HR1 and Cdc42 was much lower than expected, we reasoned that the C terminus of Cdc42 might be necessary for a high affinity interaction. The binding experiments were repeated with full-length [^3^H]GTP·Cdc42, but the affinity of the HR1 domain for full-length Cdc42 was similar to its affinity for truncated Cdc42 (*K_d_* ≈ 5 μm; Fig. 1*C*). Thus, the C-terminal region of Cdc42 is not required for maximal binding of TOCA1 HR1.

**FIGURE 2.**
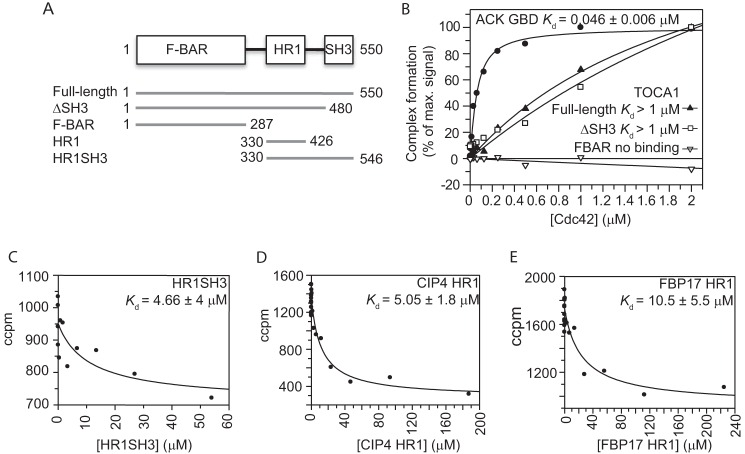
**The Cdc42-HR1 interaction is of low affinity in the context of full-length protein and in TOCA1 paralogues.** *A*, diagram illustrating the TOCA1 constructs assayed for Cdc42 binding. Domain boundaries are derived from secondary structure predictions; *B*, binding curves derived from direct binding assays, in which the indicated concentrations of Cdc42Δ7Q61L·[^3^H]GTP were incubated with 30 nm GST-ACK or His-tagged TOCA1 constructs, as indicated, in SPAs. The SPA signal was corrected by subtraction of control data with no fusion protein. The data were fitted to a binding isotherm to give an apparent *K_d_* and are expressed as a percentage of the maximum signal. *C–E*, representative examples of competition SPA experiments carried out with the indicated concentrations of the TOCA1 HR1-SH3 construct titrated into 30 nm GST-ACK and 30 nm Cdc42Δ7Q61L·[^3^H]GTP (*C*) or HR1^CIP4^ (D) or HR1^FBP17^ (*E*) titrated into 30 nm GST-ACK and 30 nm Cdc42FLQ61L·[^3^H]GTP.

Another possible explanation for the low affinities observed was that the HR1 domain alone is not sufficient for maximal binding of the TOCA proteins to Cdc42 and that the other domains are required. Indeed, GST pull-downs performed with *in vitro* translated human TOCA1 fragments had suggested that residues N-terminal to the HR1 domain may be required to stabilize the HR1 domain structure (16). Furthermore, both BAR and SH3 domains have been implicated in interactions with small G proteins (*e.g.* the BAR domain of Arfaptin2 binds to Rac1 and Arl1) (62), while an SH3 domain mediates the interaction between Rac1 and the guanine nucleotide exchange factor, β-PIX (63). TOCA1 dimerizes via its F-BAR domain, which could also affect Cdc42 binding, for example by presenting two HR1 domains for Cdc42 interactions. Various TOCA1 fragments (Fig. 2*A*) were therefore assessed for binding to full-length Cdc42 by direct SPA. The isolated F-BAR domain showed no binding to full-length Cdc42 (Fig. 2*B*). Full-length TOCA1 and ΔSH3 TOCA1 bound with micromolar affinity (Fig. 2*B*), in a similar manner to the isolated HR1 domain (Fig. 1*A*). The HR1-SH3 protein could not be purified to homogeneity as a fusion protein, so it was assayed in competition assays after cleavage of the His tag. This construct competed with GST-ACK GBD to give a similar affinity to the HR1 domain alone (*K_d_* = 4.6 ± 4 μm; Fig. 2*C*). Taken together, these data suggest that the TOCA1 HR1 domain is sufficient for maximal binding and that this binding is of a relatively low affinity compared with many other Cdc42·effector complexes.

The low affinity of the TOCA1 HR1-Cdc42 interaction raised the question of whether the other known Cdc42-binding TOCA family proteins, FBP17 (18) and CIP4 (17), also bind weakly. The HR1 domains from FBP17 and CIP4 were purified and assayed for Cdc42 binding in competition SPAs, analogous to those carried out with the TOCA1 HR1 domain. The affinities of both the FBP17 and CIP4 HR1 domains were also in the low micromolar range (10 and 5 μm, respectively) (Fig. 2, *D* and *E*), suggesting that low affinity interactions with Cdc42 are a common feature within the TOCA family.

##### Structure of the TOCA1 HR1 Domain

Because the TOCA1 HR1 domain was sufficient for maximal Cdc42-binding, we used this construct for structural studies. Initial experiments were performed with TOCA1 residues 324–426, but we observed that the N terminus was cleaved during purification to yield a new N terminus at residue 330 (data not shown). We therefore engineered a construct comprising residues 330–426 to produce the minimal, stable HR1 domain. Backbone and side chain resonances were assigned as described (54). 2,778 non-degenerate NOE restraints were used in initial structure calculations (1,791 unambiguous and 987 ambiguous), derived from three-dimensional ^15^N-separated NOESY and ^13^C-separated NOESY experiments. There were 1,845 unambiguous NOEs and 757 ambiguous NOEs after eight iterations. 100 structures were calculated in the final iteration; the 50 lowest energy structures were water-refined; and of these, the 35 lowest energy structures were analyzed. Table 1 indicates that the HR1 domain structure is well defined by the NMR data.

**TABLE 1:**
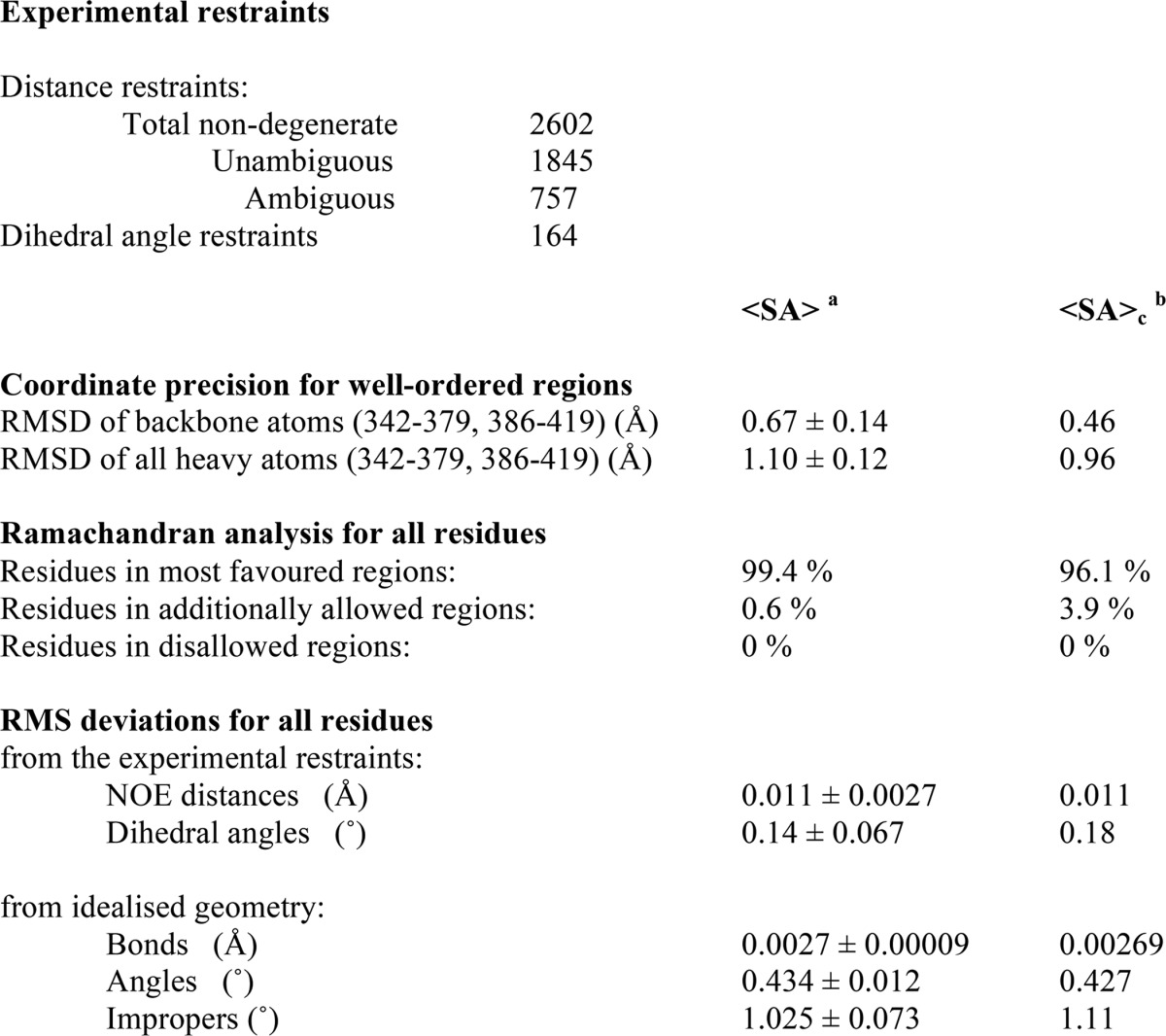
**Experimental restraints and structural statistics**

*^a^* <SA>, the average root mean square deviations for the ensemble ± S.D.

*^b^* <SA>_c_, values for the structure that is closest to the mean.

**FIGURE 3.**
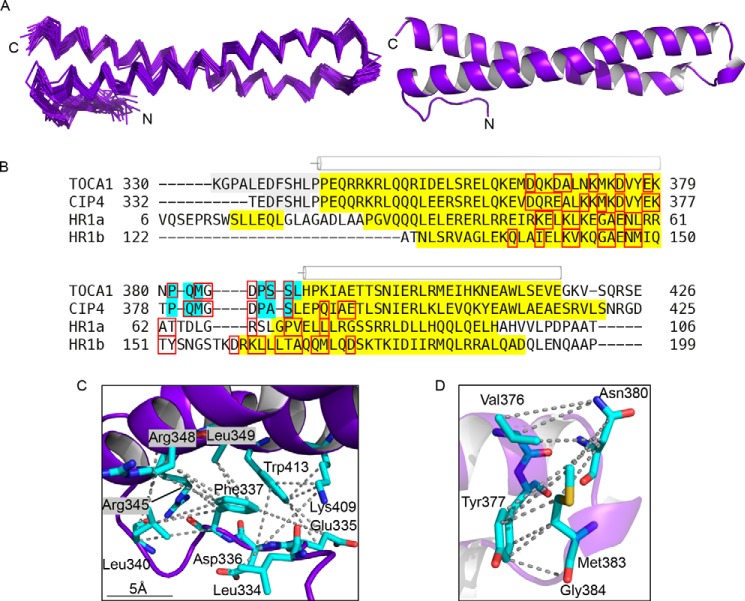
**The structure of the TOCA1 HR1 domain.** *A*, the backbone trace of the 35 lowest energy structures of the HR1 domain overlaid with the structure closest to the mean is shown alongside a schematic representation of the structure closest to the mean. Flexible regions at the N and C termini (residues 330–333 and 421–426) are omitted for clarity. *B*, a sequence alignment of the HR1 domains from TOCA1, CIP4, and PRK1. The secondary structure was deduced using Stride (64), based on the Ramachandran angles, and is indicated as follows: *gray*, turn; *yellow*, α-helix; *blue*, 3_10_ helix; *white*, coil. *C*, a *close-up* of the N-terminal region of TOCA1 HR1, indicating some of the NOEs defining its position with respect to the two α-helices. *Dotted lines*, NOE restraints. *D*, a *close-up* of the interhelix loop region showing some of the contacts between the loop and helix 1. NOEs are indicated with *dotted lines*. All structural figures were generated using PyMOL.

The structure closest to the mean is shown in Fig. 3*A*. The two α-helices of the HR1 domain interact to form an anti-parallel coiled-coil with a slight left-handed twist, reminiscent of the HR1 domains of CIP4 (47) (PDB code 2KE4) and PRK1 (42, 43) (PDB codes 1CXZ and 1URF). A sequence alignment illustrating the secondary structure elements of the TOCA1 and CIP4 HR1 domains and the HR1a and HR1b domains from PRK1 is shown in Fig. 3*B*.

In the HR1a domain of PRK1, a region N-terminal to helix 1 forms a short α-helix, which packs against both helices of the HR1 domain (42). This region of TOCA1 HR1 (residues 334–340) is well defined in the family of structures (Fig. 3*A*) but does not form an α-helix. It instead forms a series of turns, defined by NOE restraints observed between residues separated by one (residues 332–334, 333–335, etc.) or two (residues 337–340) residues in the sequence and the φ and ψ angles, assessed using Stride (64). These turns cause the chain to reverse direction, allowing the N-terminal segment (residues 334–340) to contact both helices of the HR1 domain. Long range NOEs were observed linking Leu-334, Glu-335, and Asp-336 with Trp-413 of helix 2, Leu-334 with Lys-409 of helix 2, and Phe-337 and Ser-338 with Arg-345, Arg-348, and Leu-349 of helix 1. These contacts are summarized in Fig. 3*C*.

The two α-helices of TOCA1 HR1 are separated by a long loop of 10 residues (residues 380–389) that contains two short 3_10_ helices (residues 381–383 and 386–389). Interestingly, side chains of residues within the loop region point back toward helix 1; for example, there are numerous distinct NOEs between the side chains of Asn-380 and Met-383 of the loop region and Tyr-377 and Val-376 of helix 1 (Fig. 3*D*). The backbone NH and CHα groups of Gly-384 and Asp-385 also show NOEs with the side chain of Tyr-377.

##### Mapping the TOCA1 and Cdc42 Binding Interfaces

**FIGURE 4.**
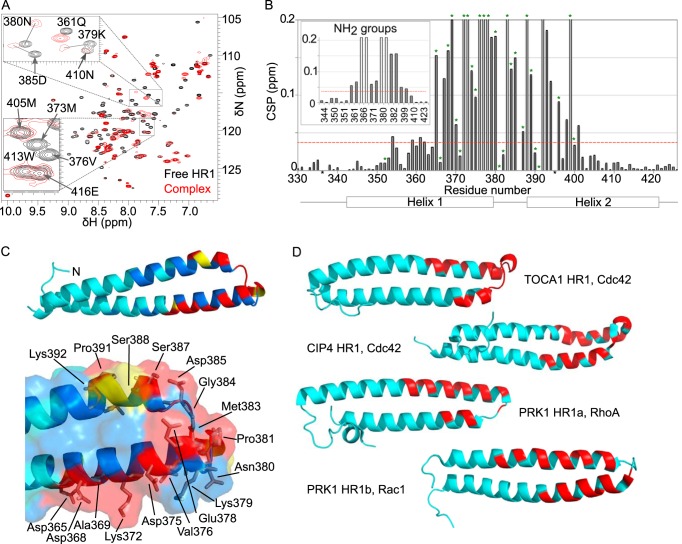
**Mapping the binding surface of Cdc42 onto the TOCA1 HR1 domain.** *A*, the ^15^N HSQC of 200 μm TOCA1 HR1 domain is shown in the free form (*black*) and in the presence of a 4-fold molar excess of Cdc42Δ7Q61L·GMPPNP (*red*). Expansions of two regions are shown with peak assignments, showing backbone amides in fast or intermediate exchange. *B*, CSPs were calculated as described under “Experimental Procedures” and are shown for backbone and side chain NH groups. The mean CSP is marked with a *red line*. Residues that disappeared in the presence of Cdc42 were assigned a CSP of 0.2 but were excluded when calculating the mean CSP and are indicated with *open bars*. Those that were not traceable due to spectral overlap were assigned a CSP of zero and are marked with an *asterisk below* the *bar*. Residues with affected side chain CSPs derived from ^13^C HSQCs are marked with *green asterisks above* the *bars*. Secondary structure elements are shown *below* the *graph. C*, a *schematic representation* of the HR1 domain. Residues with significantly affected backbone or side chain chemical shifts when Cdc42 bound and that are buried are *colored dark blue*, whereas those that are solvent-accessible are *colored yellow*. Residues with significantly affected backbone and side chain groups that are solvent-accessible are *colored red*. A *close-up* of the binding region is shown, with affected side chain heavy atoms shown as *sticks. D*, the G protein-binding region is marked in *red* onto structures of the HR1 domains as indicated.

The HR1^TOCA1^-Cdc42 interface was investigated using NMR spectroscopy. A series of ^15^N HSQC experiments was recorded on ^15^N-labeled TOCA1 HR1 domain in the presence of increasing concentrations of unlabeled Cdc42Δ7Q61L·GMPPNP to map the Cdc42-binding surface. A comparison of the ^15^N HSQC spectra of free HR1 and HR1 in the presence of excess Cdc42 shows that although some peaks were shifted, several were much broader in the complex, and a considerable subset had disappeared (Fig. 4*A*). This behavior cannot be explained by the increase in molecular mass (from 12 to 33 kDa) when Cdc42 binds and is more likely to be due to conformational exchange. This leads to broadening of the peaks so that they are not detectable. Overall chemical shift perturbations (CSPs) were calculated for each residue, whereas those that had disappeared were assigned a shift change of 0.2 (Fig. 4*B*). A peak that disappeared or had a CSP above the mean CSP for the spectrum was considered to be significantly affected.

^15^N HSQC shift mapping experiments report on changes to amide groups, which are mainly inaccessible because they are buried inside the helices and are involved in hydrogen bonds. Therefore, ^13^C HSQC and methyl-selective SOFAST-HMQC (65) experiments were also recorded on ^15^N,^13^C-labeled TOCA1 HR1 to yield more information on side chain involvement. The affected CH groups underwent significant line broadening, similarly to the NH peaks. Side chains whose CH groups disappeared in the presence of Cdc42 are marked on the graph in Fig. 4*B* with *green asterisks*.

TOCA1 residues whose signals were affected by Cdc42 binding were mapped onto the structure of TOCA1 HR1 (Fig. 4*C*). The changes were localized to one end of the coiled-coil, and the binding site appeared to include residues from both α-helices and the loop region that joins them. Residues outside of this region were not significantly affected, indicating that there was no widespread conformational change. The residues in the interhelical loop and helix 1 that contact each other (Fig. 3*D*) show shift changes in their backbone NH and side chains in the presence of Cdc42. For example, the side chain of Asn-380 and the backbones of Val-376 and Tyr-377 were significantly affected but are all buried in the free TOCA1 HR1 structure, indicating that local conformational changes in the loop may facilitate complex formation. The chemical shift mapping data indicate that the G protein-binding region of the TOCA1 HR1 domain is broadly similar to that of the CIP4 and PRK1 HR1 domains (Figs. 3*B* and 4*D*).

**FIGURE 5.**
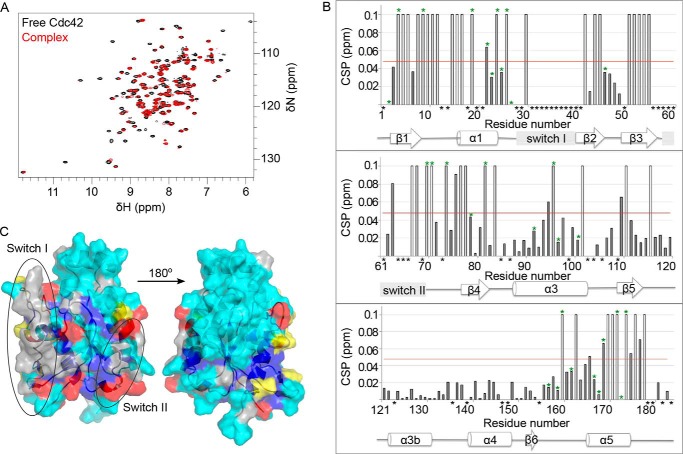
**Mapping the binding surface of the HR1 domain onto Cdc42.** *A*, the ^15^N HSQC of Cdc42Δ7Q61L·GMPPNP is shown in its free form (*black*) and in the presence of excess TOCA1 HR1 domain (1:2.2, *red*). Expansions of two regions are shown, with most peaks in fast or intermediate exchange. *B*, CSPs are shown for backbone NH groups. The *red line* indicates the mean CSP, plus one S.D. Residues that disappeared in the presence of Cdc42 were assigned a CSP of 0.1 and are indicated with *open bars*. Those that were not traceable due to overlap are marked with an *asterisk*. Residues with disappeared peaks in ^13^C HSQC experiments are marked on the chart with *green asterisks*. Secondary structure elements are indicated *below* the *graph. C*, the residues with significantly affected backbone and side chain groups are highlighted on an NMR structure of free Cdc42Δ7Q61L·GMPPNP; those that are buried are *colored dark blue*, whereas those that are solvent-accessible are *colored red*. Residues with either side chain or backbone groups affected are *colored blue* if buried and *yellow* if solvent-accessible. Residues without information from shift mapping are *colored gray*. The flexible switch regions are *circled*.

The corresponding ^15^N and ^13^C NMR experiments were also recorded on ^15^N-Cdc42Δ7Q61L·GMPPNP or ^15^N/^13^C -Cdc42Δ7Q61L·GMPPNP in the presence of unlabeled HR1 domain. The overall CSP was calculated for each residue. As was the case when labeled HR1 was observed, several peaks were shifted in the complex, but many disappeared, indicating exchange on an unfavorable, millisecond time scale (Fig. 5*A*). Detailed side chain data could not be obtained for all residues due to spectral overlap, but constant time ^13^C HSQC and methyl-selective SOFAST-HMQC experiments provided further information on certain well resolved side chains (marked with *green asterisks* in Fig. 5*B*).

As many of the peaks disappeared, the mean chemical shift change was relatively low, so a threshold of the mean plus one S.D. value was used to define a significant CSP. Residues that disappeared were also classed as significantly affected. Parts of the switch regions (Fig. 5, *B* and *C*) are invisible in NMR spectra recorded on free Cdc42 due to conformational exchange. These switch regions become visible in Cdc42 and other small G protein·effector complexes (46, 61, 66) due to a decrease in conformational freedom upon complex formation. The switch regions of Cdc42 did not, however, become visible in the presence of the TOCA1 HR1 domain. Indeed, Ser-30 of switch I and Arg-66, Arg-68, Leu-70, and Ser-71 of switch II are visible in free Cdc42 but disappear in the presence of the HR1 domain. This suggests that the switch regions are not rigidified in the HR1 complex and are still in conformational exchange. Nevertheless, mapping of the affected residues onto the NMR structure of free Cdc42Δ7Q61L·GMPPNP (Fig. 5*C*)^8^ shows that, although they are relatively widespread compared with changes in the HR1 domain, in general, they are on the face of the protein that includes the switches. Although the binding interface may be overestimated, this suggests that the switch regions are involved in binding to TOCA1.

##### Modeling the Cdc42·TOCA1 HR1 Complex

The Cdc42·HR1^TOCA1^ complex was not amenable to full structural analysis due to the weak interaction and the extensive exchange broadening seen in the NMR experiments. HADDOCK (67) was therefore used to perform rigid body docking based on the structures of free HR1 domain and Cdc42 and ambiguous interaction restraints derived from the titration experiments described above. Residues with significantly affected resonances and more than 50% solvent accessibility were defined as active. Passive residues were defined automatically as those neighboring active residues.

**FIGURE 6.**
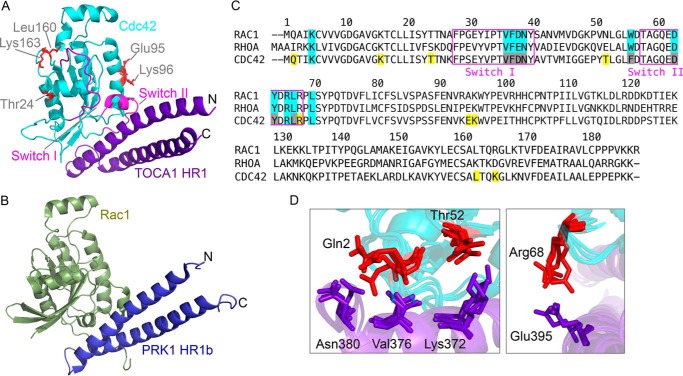
**Model of Cdc42·HR1 complex.** *A*, a representative model of the Cdc42·HR1 complex from the cluster closest to the lowest energy model produced using HADDOCK (67). Residues of Cdc42 that are affected in the presence of the HR1 domain but are not in close proximity to it are *colored* in *red* and *labeled. B*, structure of Rac1 in complex with the HR1b domain of PRK1 (46) (PDB code 2RMK). *C*, sequence alignment of RhoA, Cdc42 and Rac1. Contact residues of RhoA and Rac1 to PRK1 HR1a and HR1b, respectively, are *colored cyan*. Residues of Cdc42 that disappear or show chemical shift changes in the presence of TOCA1 are *colored cyan* if also identified as contacts in RhoA and Rac1 and *yellow* if they are not. Residues equivalent to Rac1 and RhoA contact sites but that are invisible in free Cdc42 are *gray. D*, regions of interest of the Cdc42·HR1 domain model. The four lowest energy structures in the chosen HADDOCK cluster are shown *overlaid*, with the residues of interest shown as *sticks* and *labeled*. Cdc42 is shown in *cyan*, and TOCA1 is shown in *purple*.

The orientation of the HR1 domain with respect to Cdc42 cannot be definitively concluded in the absence of unambiguous distance restraints; hence, HADDOCK produced a set of models in which the HR1 domain contacts the same surface on Cdc42 but is in various orientations with respect to Cdc42. The cluster with the lowest root mean square deviation from the lowest energy structure is assumed to be the best model. By these criteria, in the best model, the HR1 domain is in a similar orientation to the HR1a domain of PRK1 bound to RhoA and the HR1b domain bound to Rac1. A representative model from this cluster is shown in Fig. 6*A* alongside the Rac1-HR1b structure (46) (PDB code 2RMK) in Fig. 6*B*.

A sequence alignment of RhoA, Cdc42, and Rac1 is shown in Fig. 6*C*. The RhoA and Rac1 contact residues in the switch regions are invisible in the spectra of Cdc42, but they are generally conserved between all three G proteins. Several Cdc42 residues identified by chemical shift mapping are not in close contact in the Cdc42·TOCA1 model (Fig. 6*A*). Some of these can be rationalized; for example, Thr-24^Cdc42^, Leu-160^Cdc42^, and Lys-163^Cdc42^ all pack behind switch I and are likely to be affected by conformational changes within the switch, while Glu-95^Cdc42^ and Lys-96^Cdc42^ are in the helix behind switch II. Other residues that are affected in the Cdc42·TOCA1 complex but that do not correspond to contact residues of RhoA or Rac1 (Fig. 6*C*) include Gln-2^Cdc42^, Lys-16^Cdc42^, Thr-52^Cdc42^, and Arg-68^Cdc42^. Lys-16^Cdc42^ is unlikely to be a contact residue because it is involved in nucleotide binding, but the others may represent specific Cdc42-TOCA1 contacts. In the model, these side chains are involved in direct contacts (Fig. 6*D*).

##### Competition between N-WASP and TOCA1

**FIGURE 7.**
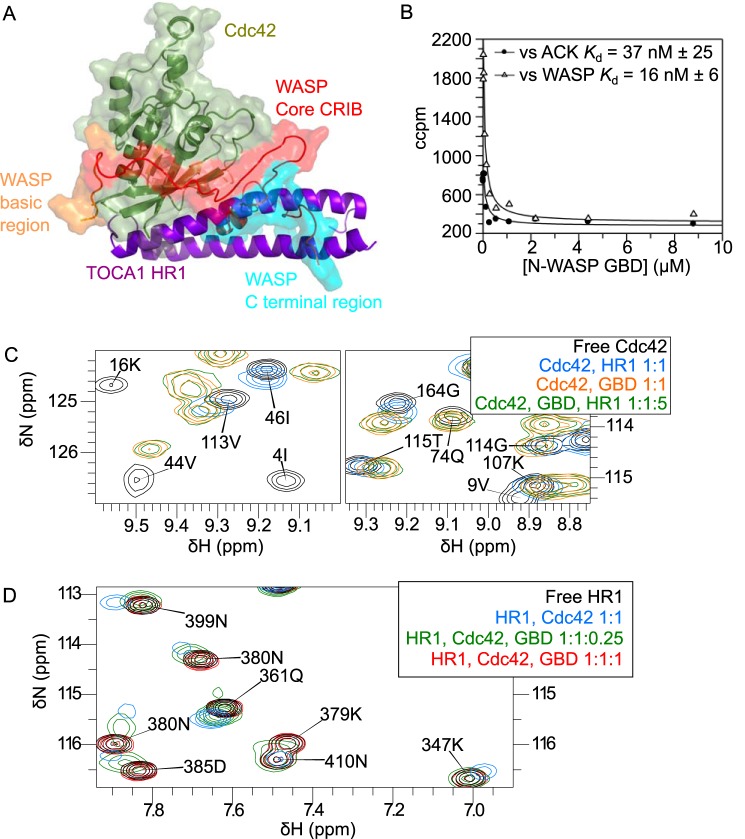
**The N-WASP GBD displaces the TOCA1 HR1 domain.** *A*, the model of the Cdc42·TOCA1 HR1 domain complex overlaid with the Cdc42-WASP structure. Cdc42 is shown in *green*, and TOCA1 is shown in *purple*. The core CRIB region of WASP is shown in *red*, whereas its basic region is shown in *orange* and the C-terminal region required for maximal affinity is shown in *cyan*. A *semitransparent surface representation* of Cdc42 and WASP is shown *overlaid* with the schematic. *B*, competition SPA experiments carried out with indicated concentrations of the N-WASP GBD construct titrated into 30 nm GST-ACK or GST-WASP GBD and 30 nm Cdc42Δ7Q61L·[^3^H]GTP. *C*, Selected regions of the ^15^N HSQC of 145 μm Cdc42Δ7Q61L·GMPPNP with the indicated ratios of the TOCA1 HR1 domain, the N-WASP GBD, or both, showing that the TOCA HR1 domain does not displace the N-WASP GBD. *D*, selected regions of the ^15^N HSQC of 600 μm TOCA1 HR1 domain in complex with Cdc42 in the absence and presence of the N-WASP GBD, showing displacement of Cdc42 from the HR1 domain by N-WASP.

From the known interactions and effects of the proteins in biological systems, it has been suggested that TOCA1 and N-WASP could bind Cdc42 simultaneously (16). Studies in CHO cells indicated that a Cdc42·N-WASP·TOCA1 complex existed (30) because FRET was observed between RFP-TOCA1 and GFP-N-WASP, and the efficiency was decreased when an N-WASP mutant was used that no longer binds Cdc42. An overlay of the HADDOCK model of the Cdc42·HR1^TOCA1^ complex and the structure of Cdc42 in complex with the GBD of the N-WASP homologue, WASP (49) (PDB code 1CEE), shows that the HR1 and GBD binding sites only partly overlap, and, therefore, a ternary complex remained possible (Fig. 7*A*). Interestingly, the presence of the TOCA1 HR1 would not prevent the core CRIB of WASP from binding to Cdc42, although the regions C-terminal to the CRIB that are required for high affinity binding of WASP (68) would interfere sterically with the TOCA1 HR1. A basic region in WASP including three lysines (residues 230–232), N-terminal to the core CRIB, has been implicated in an electrostatic steering mechanism (69), and these residues would be free to bind in the presence of TOCA1 HR1 (Fig. 7*A*).

An N-WASP GBD construct was produced, and its affinity for Cdc42 was measured by competition SPA (Fig. 7*B*). The *K_d_* that was determined (37 nm) is consistent with the previously reported affinity (69). Unlabeled N-WASP GBD was titrated into ^15^N-Cdc42Δ7Q61L·GMPPNP, and the backbone NH groups were monitored using HSQCs (Fig. 7*C*). Unlabeled HR1^TOCA1^ was then added to the Cdc42·N-WASP complex, and no changes were seen, suggesting that the N-WASP GBD was not displaced even in the presence of a 5-fold excess of HR1^TOCA1^. These experiments were recorded at sufficiently high protein concentrations (145 μm Cdc42, 145 μm N-WASP GBD, 725 μm TOCA1 HR1 domain) to be far in excess of the *K_d_* values of the individual interactions (TOCA1 *K_d_* ≈ 5 μm, N-WASP *K_d_* = 37 nm). A comparison of the HSQC experiments recorded on ^15^N-Cdc42 alone, in the presence of TOCA1 HR1, N-WASP GBD, or both, shows that the spectra in the presence of N-WASP and in the presence of both N-WASP and TOCA1 HR1 are identical (Fig. 7*C*).

Furthermore, ^15^N-TOCA1 HR1 was monitored in the presence of unlabeled Cdc42Δ7Q61L·GMPPNP (1:1) before and after the addition of 0.25 and 1.0 eq of unlabeled N-WASP GBD. The spectrum when N-WASP and TOCA1 were equimolar was identical to that of the free HR1 domain, whereas the spectrum in the presence of 0.25 eq of N-WASP was intermediate between the TOCA1 HR1 free and complex spectra (Fig. 7*D*). When in fast exchange, the NMR signal represents a population-weighted average between free and bound states, so the intermediate spectrum indicates that the population comprises a mixture of free and bound HR1 domain. Hence, a third, intermediate state that includes all three proteins is unlikely. Again, the experiments were recorded on protein samples far in excess of the individual *K_d_* values (600 μm each protein). These data indicate that the HR1 domain is displaced from Cdc42 by N-WASP and that a ternary complex comprising TOCA1 HR1, N-WASP GBD, and Cdc42 is not formed. Taken together, the data in Fig. 7, *C* and *D*, indicate unidirectional competition for Cdc42 binding in which the N-WASP GBD displaces TOCA1 HR1 but not *vice versa*.

To extend these studies to a more complex system and to assess the ability of TOCA1 HR1 to compete with full-length N-WASP, pyrene actin assays were employed. These assays, described in detail elsewhere (59), were carried out using pyrene actin-supplemented *Xenopus* extracts into which exogenous TOCA1 HR1 domain or N-WASP GBD was added, to assess their effects on actin polymerization. Actin polymerization in all cases was initiated by the addition of PI(4,5)P_2_-containing liposomes. Actin polymerization triggered by the addition of PI(4,5)P_2_-containing liposomes has previously been shown to depend on TOCA1 and N-WASP (32). Endogenous N-WASP is present at ∼100 nm in *Xenopus* extracts, whereas TOCA1 is present at a 10-fold lower concentration than N-WASP (16).

**FIGURE 8.**
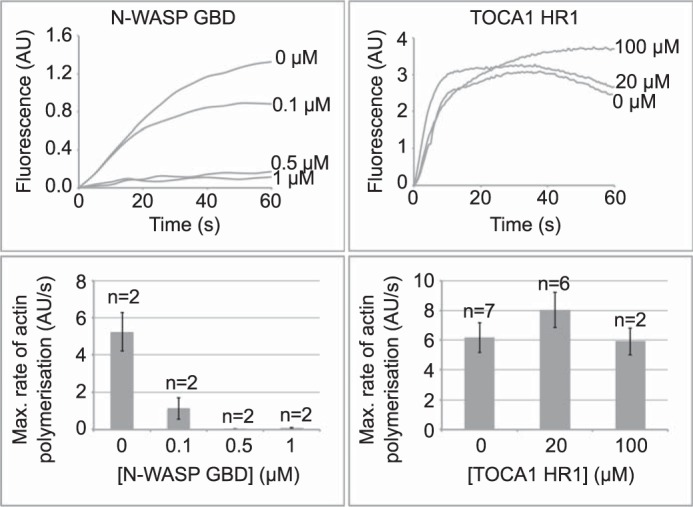
**Actin polymerization downstream of Cdc42·N-WASP·TOCA1 is inhibited by excess N-WASP GBD but not by the TOCA1 HR1 domain.** Fluorescence curves show actin polymerization in the presence of increasing concentrations of N-WASP GBD or TOCA1 HR1 domain as indicated. Maximal rates of actin polymerization derived from the linear region of the curves are represented in *bar charts below. Error bars*, S.E.

The addition of the isolated N-WASP GBD significantly inhibited the polymerization of actin at concentrations as low as 100 nm and completely abolished polymerization at higher concentrations (Fig. 8). The GBD presumably acts as a dominant negative, sequestering endogenous Cdc42 and preventing endogenous full-length N-WASP from binding and becoming activated. The addition of the TOCA1 HR1 domain to 100 μm had no significant effect on the rate of actin polymerization or maximum fluorescence. This is consistent with endogenous N-WASP, activated by other components of the assay, outcompeting the TOCA1 HR1 domain for Cdc42 binding.

## Discussion

### 

#### 

##### The Cdc42-TOCA1 Interaction

The TOCA1 HR1 domain alone is sufficient for Cdc42 binding *in vitro*, yet the affinity of the TOCA1 HR1 domain for Cdc42 is remarkably low (*K_d_* ≈ 5 μm). This is over 100 times lower than that of the N-WASP GBD (*K_d_* = 37 nm) and considerably lower than other known G protein-HR1 domain interactions. The polybasic tract within the C-terminal region of Cdc42 does not appear to be required for binding to TOCA1, which is in contrast to the interaction between Rac1 and the HR1b domain of PRK1 but more similar to the PRK1 HR1a-RhoA interaction. A single binding interface on both the HR1 domain and Cdc42 can be concluded from the data presented here. Furthermore, the interfaces are comparable with those of other G protein-HR1 interactions (Fig. 4), and the lowest energy model produced in rigid body docking resembles previously studied G protein·HR1 complexes (Fig. 6). It seems, therefore, that the interaction, despite its relatively low affinity, is specific and sterically similar to other HR1 domain-G protein interactions.

The TOCA1 HR1 domain is a left-handed coiled-coil comparable with other known HR1 domains (42, 43, 47). A short region N-terminal to the coiled-coil exhibits a series of turns and contacts residues of both helices of the coiled-coil (Fig. 3). The corresponding sequence in CIP4 also includes a series of turns but is flexible, whereas in the HR1a domain of PRK1, the equivalent region adopts an α-helical structure that packs against the coiled-coil. The contacts between the N-terminal region and the coiled-coil are predominantly hydrophobic in both cases, but sequence-specific contacts do not appear to be conserved. This region is distant from the G protein-binding interface of the HR1 domains, so the structural differences may relate to the structure and regulation of these domains rather than their G protein interactions.

The interhelical loops of TOCA1 and CIP4 differ from the same region in the HR1 domains of PRK1 in that they are longer and contain two short stretches of 3_10_-helix. This region lies within the G protein-binding surface of all of the HR1 domains (Fig. 4*D*). TOCA1 and CIP4 both bind weakly to Cdc42, whereas the HR1a domain of PRK1 binds tightly to RhoA and Rac1, and the HR1b domain binds to Rac1. The structural features shared by TOCA1 and CIP4 may therefore be related to Cdc42 binding specificity and the low affinities. In free TOCA1, the side chains of the interhelical region make extensive contacts with residues in helix 1. Many of these residues are significantly affected in the presence of Cdc42, so it is likely that the conformation of this loop is altered in the Cdc42 complex. These observations therefore provide a molecular mechanism whereby mutation of Met^383^-Gly^384^-Asp^385^ to Ile^383^-Ser^384^-Thr^385^ abolishes TOCA1 binding to Cdc42 (16).

The lowest energy model produced by HADDOCK using ambiguous interaction restraints from the titration data resembled the NMR structures of RhoA and Rac1 in complex with their HR1 domain partners (42, 46). Some speculative conclusions can be made based on this model. For example, Phe-56^Cdc42^, which is not visible in free Cdc42 or Cdc42·HR1^TOCA1^, is close to the TOCA1 HR1 (Fig. 6*A*). Phe-56^Cdc42^, which is a Trp in both Rac1 and RhoA (Fig. 6*C*), is thought to pack behind switch I when Cdc42 interacts with ACK, maintaining the switch in a binding-competent orientation (70). This residue has also been identified as important for Cdc42-WASP binding (71). Phe-56^Cdc42^ is therefore likely to be involved in the Cdc42-TOCA1 interaction, probably by stabilizing the position of switch I.

Some residues that are affected in the Cdc42·HR1^TOCA1^ complex but do not correspond to contact residues of RhoA or Rac1 (Fig. 6*C*) may contact HR1^TOCA1^ directly (Fig. 6*D*). Gln-2^Cdc42^, which has also been identified as a contact residue in the Cdc42·ACK complex (61), contacts Val-376^TOCA1^ and Asn-380^TOCA1^ in the model and disrupts the contacts between the interhelical loop and the first helix of the TOCA1 coiled-coil. Thr-52^Cdc42^, which has also been identified as making minor contacts with ACK (61, 72), falls near the side chains of HR1^TOCA1^ helix 1, particularly Lys-372^TOCA1^, whereas the equivalent position in Rac1 is Asn-52^Rac1^. N52T is one of a combination of seven residues found to confer ACK binding on Rac1 (72) and so may represent a specific Cdc42-effector contact residue. The position equivalent to Lys-372^TOCA1^ in PRK1 is Glu-58^HR1a^ or Gln-151^HR1b^. Thr-52^Cdc42^-Lys-372^TOCA1^ may therefore represent a specific Cdc42-HR1^TOCA1^ contact. Arg-68^Cdc42^ of switch II is positioned close to Glu-395^TOCA1^ (Fig. 6*D*), suggesting a direct electrostatic contact between switch II of Cdc42 and helix 2 of the HR1 domain. The equivalent Arg in Rac1 and RhoA is pointing away from the HR1 domains of PRK1. The importance of this residue in the Cdc42-TOCA1 interaction remains unclear, although its mutation reduces binding to RhoGAP, suggesting that it can be involved in Cdc42 interactions (53).

The solution structure of the TOCA1 HR1 domain presented here, along with the model of the HR1^TOCA1^·Cdc42 complex is consistent with a conserved mode of binding across the known HR1 domain-Rho family interactions, despite their differing affinities. The weak binding prevented detailed structural and thermodynamic studies of the complex. Nonetheless, structural studies of the TOCA1 HR1 domain, combined with chemical shift mapping, have highlighted some potentially interesting differences between Cdc42-HR1^TOCA1^ and RhoA/Rac1-HR1^PRK1^ binding.

We have previously postulated that the inherent flexibility of HR1 domains contributes to their ability to bind to different Rho family G proteins, with Rho-binding HR1 domains displaying increased flexibility, reflected in their lower melting temperatures (*T_m_*) and Rac binders being more rigid (44, 45). The *T_m_* of the TOCA1 HR1 domain is 61.9 °C (data not shown), which is the highest *T_m_* that we have measured for an HR1 domain thus far. As such, the ability of the TOCA1 HR1 domain to bind to Cdc42 (a close relative of Rac1 rather than RhoA) fits this trend. An investigation into the local motions, particularly in the G protein-binding regions, may offer further insight into the differential specificities and affinities of G protein-HR1 domain interactions.

##### Significance of a Weak, Transient Interaction

The low affinity of the Cdc42-HR1^TOCA1^ interaction is consistent with a tightly spatially and temporally regulated pathway, requiring combinatorial signals leading to a series of coincident weak interactions that elicit full activation. The HR1 domains from other TOCA family members, CIP4 and FBP17, also bind at low micromolar affinities to Cdc42, so the low affinity interaction appears to be commonplace among this family of HR1 domain proteins, in contrast to the PRK family. Weak, transient protein-protein interactions are functionally significant in several systems (73–75); for example, the binding of adaptor proteins to protein cargo during the formation of clathrin-coated vesicles in endocytosis involves multiple interactions of micromolar affinity (76, 77).

The low affinity of the HR1^TOCA1^-Cdc42 interaction in the context of the physiological concentration of TOCA1 in *Xenopus* extracts (∼10 nm) (16) suggests that binding between TOCA1 and Cdc42 is likely to occur *in vivo* only when TOCA1 is at high local concentrations and membrane-localized and therefore in close proximity to activated Cdc42. Evidence suggests that the TOCA family of proteins are recruited to the membrane via an interaction between their F-BAR domain and specific signaling lipids. For example, electrostatic interactions between the F-BAR domain and the membrane are required for TOCA1 recruitment to membrane vesicles and tubules (27), and TOCA1-dependent actin polymerization is known to depend specifically on PI(4,5)P_2_ (32). Furthermore, the isolated F-BAR domain of FBP17 has been shown to induce membrane tubulation of brain liposomes and BAR domain proteins that promote tubulation cluster on membranes at high densities (33). Once at the membrane, high local concentrations of TOCA1 could exceed the *K_d_* of F-BAR dimerization (likely to be comparable with that of the FCHo2 F-BAR domain (2.5 μm) (41)) and that of the Cdc42-HR1^TOCA1^ interaction. Cdc42-HR1^TOCA1^ binding would then be favorable, as long as coincident activation of Cdc42 had occurred, leading to stabilization of TOCA1 at the membrane and downstream activation of N-WASP.

It has been postulated that WASP and N-WASP exist in equilibrium between folded (inactive) and unfolded (active) forms, and the affinity of Cdc42 for the unfolded WASP proteins is significantly enhanced (78). The unfolded, high affinity state of WASP is represented by a short peptide, the GBD, which binds with a low nanomolar affinity to Cdc42 (49). In contrast, the best estimate of the affinity of full-length WASP for Cdc42 is low micromolar (79). In the inactive state of WASP, the actin- and Arp2/3-binding VCA domain contacts the GBD (21, 23, 80), competing for Cdc42 binding. The high affinity of Cdc42 for the unfolded, active form pushes the equilibrium in favor of (N-)WASP activation. Binding of PI(4,5)P_2_ to the basic region just N-terminal to the GBD further favors the active conformation (21). A substantial body of data has illuminated the complex regulation of WASP/N-WASP proteins, and current evidence suggests that these allosteric activation mechanisms and oligomerization combine to regulate WASP activity, allowing the synchronization and integration of multiple potential activation signals (reviewed in Ref. 24). Our data are easily reconciled with this model.

We envisage that TOCA1 is first recruited to the appropriate membrane in response to PI(4,5)P_2_ via its F-BAR domain, where the local increase in concentration favors F-BAR-mediated dimerization of TOCA1. Cdc42 is activated in response to co-incident signals and can then bind to TOCA1, further stabilizing TOCA1 at the membrane. TOCA1 can then recruit N-WASP (26) via an interaction between its SH3 domain and the N-WASP proline-rich region (16). The recruitment of N-WASP alone and of the N-WASP·WIP complex by TOCA1 and FBP17 has been demonstrated (26). WIP inhibits the activation of N-WASP by Cdc42, an effect that is reversed by TOCA1 (16). It may therefore be envisaged that WIP and TOCA1 exert opposing allosteric effects on N-WASP, with TOCA1 favoring the unfolded, active conformation of N-WASP and increasing its affinity for Cdc42. TOCA1 may also activate N-WASP by effective oligomerization because clustering of TOCA1 at the membrane following coincident interactions with PI(4,5)P_2_ and Cdc42 would in turn lead to clustering of N-WASP, in addition to pushing the equilibrium toward the unfolded, active state.

In a cellular context, full-length TOCA1 and N-WASP are likely to have similar affinities for active Cdc42, but in the unfolded, active conformation, the affinity of N-WASP for Cdc42 dramatically increases. Our binding data suggest that TOCA1 HR1 binding is not allosterically regulated, and our NMR data, along with the high stability of TOCA1 HR1, suggest that there is no widespread conformational change in the presence of Cdc42. As full-length TOCA1 and the isolated HR1 domain bind Cdc42 with similar affinities, the N-WASP-Cdc42 interaction will be favored because the N-WASP GBD can easily outcompete the TOCA1 HR1 for Cdc42. A combination of allosteric activation by PI(4,5)P_2_, activated Cdc42 and TOCA1, and oligomeric activation implemented by TOCA1 would lead to full activation of N-WASP and downstream actin polymerization.

In such an array of molecules localized to a discrete region of the membrane, it is plausible that WASP could bind to a second Cdc42 molecule rather than displacing TOCA1 from its cognate Cdc42. Our NMR and affinity data, however, are consistent with displacement of the TOCA1 HR1 by the N-WASP GBD. Furthermore, TOCA1 is required for Cdc42-mediated activation of N-WASP·WIP (16), implying that it may not be possible for Cdc42 to bind and activate N-WASP prior to TOCA1-Cdc42 binding. The commonly used MGD → IST (Cdc42-binding deficient) mutant of TOCA1 has a reduced ability to activate the N-WASP·WIP complex (26), further indicating the importance of the Cdc42-HR1^TOCA1^ interaction prior to downstream activation of N-WASP.

In light of this, we favor an “effector handover” scheme whereby TOCA1 interacts with Cdc42 prior to N-WASP activation, after which N-WASP displaces TOCA1 from its bound Cdc42 in order to be fully activated rather than binding a second Cdc42 molecule. Potentially, the TOCA1-Cdc42 interaction functions to position N-WASP and Cdc42 such that they are poised to interact with high affinity. The concomitant release of TOCA1 from Cdc42 while still bound to N-WASP presumably enhances the ability of TOCA1 to further activate N-WASP·WIP-induced actin polymerization. There is an advantage to such an effector handover, in that N-WASP would only be robustly recruited when F-BAR domains are already present. Hence, actin polymerization cannot occur until F-BAR domains are poised for membrane distortion.

**FIGURE 9.**
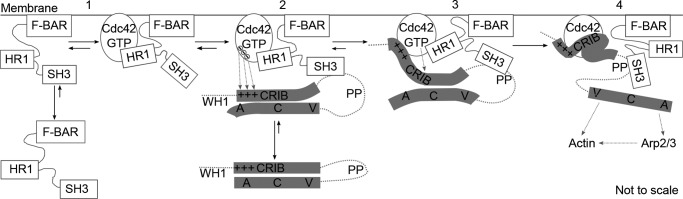
**A simplified model of the early stages of Cdc42·N-WASP·TOCA1-dependent actin polymerization.** *Step 1*, TOCA1 is recruited to the membrane via its F-BAR domain and/or Cdc42 interactions. F-BAR oligomerization is expected to occur following membrane binding, but a single monomer is shown for clarity. *Step 2*, N-WASP exists in an inactive, folded conformation. The TOCA1 SH3 domain interacts with N-WASP, causing an activatory allosteric effect. The HR1^TOCA1^-Cdc42 and SH3^TOCA1^-N-WASP interactions position Cdc42 and N-WASP for binding. *Step 3*, electrostatic interactions between Cdc42 and the basic region upstream of the CRIB initiate Cdc42·N-WASP binding. *Step 4*, the core CRIB binds with high affinity while the region C-terminal to the CRIB displaces the TOCA1 HR1 domain and increases the affinity of the N-WASP-Cdc42 interaction further. The VCA domain is released for downstream interactions, and actin polymerization proceeds. *WH1*, WASP homology 1 domain; *PP*, proline-rich region; *VCA*, verprolin homology, cofilin homology, acidic region.

Our model of the Cdc42·HR1^TOCA1^ complex indicates a mechanism by which such a handover could take place (Fig. 9) because it shows that the effector binding sites only partially overlap on Cdc42. The lysine residues thought to be involved in an electrostatic steering mechanism in WASP-Cdc42 binding (69) are conserved in N-WASP and would be able to interact with Cdc42 even when the TOCA1 HR1 domain is already bound. It has been postulated that the initial interactions between this basic region and Cdc42 could stabilize the active conformation of WASP, leading to high affinity binding between the core CRIB and Cdc42 (68). The region C-terminal to the core CRIB, required for maximal affinity binding (68), would then fully displace the TOCA1 HR1.

In conclusion, the data presented here show that the TOCA1 HR1 domain is sufficient for Cdc42 binding *in vitro* and that the interaction is of micromolar affinity, lower than that of other G protein-HR1 domain interactions. The analogous HR1 domains from other TOCA1 family members, FBP17 and CIP4, also exhibit micromolar affinity for Cdc42. A role for the TOCA1-, FBP17-, and CIP4-Cdc42 interactions in the recruitment of these proteins to the membrane therefore appears unlikely. Instead, our findings agree with earlier suggestions that the F-BAR domain is responsible for membrane recruitment (27, 33). The role of the Cdc42-TOCA1 interaction remains somewhat elusive, but it may serve to position activated Cdc42 and N-WASP to allow full activation of N-WASP and as such serve to couple F-BAR-mediated membrane deformation with N-WASP activation. We envisage a complex interplay of equilibria between free and bound, active and inactive Cdc42, TOCA family, and WASP family proteins, facilitating a tightly spatially and temporally regulated pathway requiring numerous simultaneous events in order to achieve appropriate and robust activation of the downstream pathway. Our data are therefore easily reconciled with the dynamic instability models described in relation to the formation of endocytic vesicles (75) and with the current data pertaining to the complex activation of WASP/N-WASP pathways by allosteric and oligomeric effects (24).

It is clear from the data presented here that TOCA1 and N-WASP do not bind Cdc42 simultaneously and that N-WASP is likely to outcompete TOCA1 for Cdc42 binding. We therefore postulate an effector handover mechanism based on current evidence surrounding WASP/N-WASP activation and our model of the Cdc42·HR1^TOCA1^ complex. The displacement of the TOCA1 HR1 domain from Cdc42 by N-WASP may represent a unidirectional step in the pathway of Cdc42·N-WASP·TOCA1-dependent actin assembly.

## Author Contributions

J. R. W. generated constructs and proteins, set up NMR experiments, analyzed NMR data, and performed binding experiments; D. N. set up NMR experiments; H. M. F. generated longer TOCA clones and proteins; J. L. G. supervised the pyrene actin assays; D. O. supervised the protein binding assays; and H. R. M. performed NMR experiments and analyzed NMR data. J. R. W., D. O., and H. R. M. wrote the paper with input from all authors.

## Footnotes

7 D. Owen, unpublished data.

8 H. R. Mott and D. Owen, unpublished data.

## Footnotes

6 The abbreviations used are:

PRK: protein kinase C related kinase
WASP: Wiskott-Aldrich syndrome protein
TOCA: transducer of Cdc42-dependent actin assembly protein
N-WASP: neural Wiskott-Aldrich syndrome protein
PI(4,5)P_2_: phosphatidylinositol 4,5-bisphosphate
HR1: homology region 1
F-BAR: Fes/CIP4 homology BAR
SH3: Src homology 3
CRIB: Cdc42- and Rac-interactive binding
CIP4: Cdc42-interacting protein 4
MBP: maltose-binding protein
GBD: G protein binding domain
SPA: scintillation proximity assay
PAK: p21-activated kinase
ACK: activated Cdc42-associated kinase
HSQC: heteronuclear single quantum correlation
GMPPNP: guanosine 5′-[β,γ-imido] triphosphate
GTPγS: guanosine 5′-3-*O*-(thio)triphosphate
GBD: G protein-binding domain
CSP: chemical shift perturbation
PDB: Protein Data Bank.
